# Integrated proteomic and phosphoproteomic data-independent acquisition data evaluate the personalized drug responses of primary and metastatic tumors in colorectal cancer

**DOI:** 10.52601/bpr.2022.210048

**Published:** 2023-04-30

**Authors:** Xumiao Li, Yiming Huang, Kuo Zheng, Guanyu Yu, Qinqin Wang, Lei Gu, Jingquan Li, Hui Wang, Wei Zhang, Yidi Sun, Chen Li

**Affiliations:** 1 Center for Single-Cell Omics, School of Public Health, Shanghai Jiao Tong University School of Medicine, Shanghai 200025, China; 2 Institute of Neuroscience, CAS Center for Excellence in Brain Science and Intelligence Technology, Chinese Academy of Sciences, Shanghai 200031, China; 3 Colorectal Surgery Department, Changhai Hospital, Naval Medical University, Shanghai 200433, China

**Keywords:** Proteomics, Phosphoproteomics, Data-independent acquisition (DIA), Mini patient derived xenograft (MiniPDX), Kinase-substrate network, Personalized drug responses

## Abstract

Mass spectrometry (MS)-based proteomics and phosphoproteomics are powerful methods to study the biological mechanisms, diagnostic biomarkers, prognostic analysis, and drug therapy of tumors. Data-independent acquisition (DIA) mode is considered to perform better than data-dependent acquisition (DDA) mode in terms of quantitative reproducibility, specificity, accuracy, and identification of low-abundance proteins. Mini patient derived xenograft (MiniPDX) model is an effective model to assess the response to antineoplastic drugs *in vivo* and is helpful for the precise treatment of cancer patients. Kinases are favorable spots for tumor-targeted drugs, and their functional completion relies on signaling pathways through phosphorylating downstream substrates. Kinase-phosphorylation networks or edge interactions are considered more credible and permanent for characterizing complex diseases. Here, we provide a workflow for personalized drug response assessment in primary and metastatic colorectal cancer (CRC) tumors using DIA proteomic data, DIA phosphoproteomic data, and MiniPDX models. Three kinase inhibitors, afatinib, gefitinib, and regorafenib, are tested pharmacologically. The process mainly includes the following steps: clinical tissue collection, sample preparation, hybrid spectral libraries establishment, MS data acquisition, kinase-substrate network construction, *in vivo* drug test, and elastic regression modeling. Our protocol gives a more direct data basis for individual drug responses, and will improve the selection of treatment strategies for patients without the druggable mutation.

## INTRODUCTION

Proteomics is a powerful method to study the biological mechanisms, diagnostic biomarkers, prognostic analysis, and drug therapy of tumors. Considering the important role of phosphorylation on cellular signal transduction and regulation, phosphoproteomics is an important complementary approach to proteomics (Surmen* et al.* 2020). Mass spectrometry (MS) has become an important tool for comprehensive proteomic analysis (Aebersold and Mann 2016). Up to now, there are two main modes of MS acquisition data, termed data-dependent acquisition (DDA) and data-independent acquisition (DIA). Unlike the DDA mode, which selects the most abundant precursor ions in the MS1 scan for fragmentation and acquires the corresponding MS2 spectra, the DIA mode circularly fragments all possible generated precursor ions through the predefined *m*/*z* windows and acquires the MS2 spectra throughout the measurement scanning range (Bruderer* et al.* 2015; Gillet* et al.* 2012; Sajic* et al.* 2015). Compared with DDA mode, DIA mode was superior in terms of quantitative reproducibility, specificity, and accuracy (Barkovits* et al.* 2020; Ludwig* et al.* 2018). Moreover, DIA-based proteomics was found to be superior to DDA-based proteomics analysis in identifying and qualifying low-abundance proteins (Barkovits* et al.* 2020; Gotti* et al.* 2021). There have been many DIA mode proteomics analyses for clinical studies (Krasny and Huang 2021), especially suitable for cohorts with large sample sizes (Bader* et al.* 2020; Li* et al.* 2020; Meier-Abt* et al.* 2021).

However, compared with DDA, the analysis of fragment ion spectra generated in DIA mode requires more elaborate algorithms (Bilbao* et al.* 2015). An important prerequisite for DIA proteomic data analysis is to construct a suitable spectral library for peptide identification and quantification (Barkovits* et al.* 2020). Project-specific spectral library is usually constructed based on peptide identification of conventional DDA mode to perform protein identification and quantification of raw DIA data (Lou* et al.* 2020; Schubert* et al.* 2015). Some studies (Gao* et al.* 2021; Tsou* et al.* 2016) also directly retrieve the raw data of DIA based on FASTA sequence database. Several studies have also tried new methods of generating spectral libraries. For example, Lou *et al*. (Lou* et al.* 2020) reported the construction of a hybrid spectral library that supplements a DIA experiment-derived library with a protein family-targeted virtual library predicted by deep learning. And protein identification increased by 37%–87% and peptide identification increased by 58%–161% in mouse brain tissue using this DIA hybrid library. Willems *et al*. (Willems* et al.* 2021) also reported a hybrid library generation workflow for DIA analysis, which relied on the use of data-dependent and in *silico*-predicted spectral libraries, and the results showed a significant increase in the detection of peptides. In addition to proteomics, Kitata *et al*. (Kitata* et al.* 2021) developed a global phosphoproteomics strategy based on DIA and hybrid spectral libraries derived from DDA and DIA data. Here, considering the significant individual variability among different cases and different tissue types within individuals, we provide an approach to generate a hybrid spectral library using DDA and DIA data. Project-specific library in DDA mode is deepened by repeatedly injected individual samples or sample mixture, and offline pre-fractionated peptide mixtures from pooled individual samples. Meanwhile, high-quality and high-precision spectral libraries were also directly generated from multiple DIA data.

Evaluating the personalized drug response of tumors is crucial for the precise treatment of cancer patients. Patient derived xenograft (PDX) has emerged as a powerful model for assessing antitumor drug response *in vivo* (Bertotti* et al.* 2011; Bihani* et al.* 2017; Gao* et al.* 2015; Ricci* et al.* 2014). By directly implanting patient tumor tissue or cells into immunodeficient or humanized mice, PDX models can create an environment that allows for the natural growth of cancer. It is superior to cell lines or genetically engineered mouse models because of its molecular and histopathological characteristics of original tumors. However, PDX also has some drawbacks that limit its application, such as the long time required for tumor xenograft implantation and the low engraftment rate in mouse models (Wang* et al.* 2021; Zhang* et al.* 2018). MiniPDX is a rapid, systematic method for *in vivo* drug sensitivity assay. In brief, tumor cells derived from patients are filled into capsules and then implanted into mice via a small skin incision (Zhan* et al.* 2018; Zhang* et al.* 2018). The histopathological and immunohistochemical characteristics of the tumor cells in the MiniPDX model were similar to those in the PDX model, and the time taken for MiniPDX was significantly shortened compared to PDX (Zhang* et al.* 2018; Zhao* et al.* 2018).

Kinases have been considered as favourable spots for targeted drugs because of their conservation in secondary structure elements (Manley* et al.* 2004). In spite of the role of kinases in many complex diseases (Chen* et al.* 2007; Hochgräfe* et al.* 2010; Speers* et al.* 2009; Weitsman* et al.* 2014), the accomplishment of their function relies on signalling pathways through phosphorylating downstream substrates. In other words, compared with a single kinase molecule, the kinase**-**phosphorylation network or edge interaction constituted by these molecules is considered to be more credible and permanent for characterizing complex diseases. To better characterize the kinase-phosphorylation network in colorectal cancer and explore how they are correlated to drug response, we applied a recent study where kinase-substrate networks could be constructed for every single sample using multi-omics data (Sun* et al.* 2020), and thus could be used as edge biomarkers for downstream analyses. To explore the role of the kinase-substrate network in the drug sensitivity of colorectal cancer, we constructed an elastic net to identify key edge biomarkers in response to tested drugs. A problem with linear regression is that estimated coefficients of the model can become large, making the model sensitive to inputs and overfitting. The overfitting problem often occurs in datasets with few observations (samples, *n*) and a large number of variables (*p*) (so-called *p* >> *n* problems). By including both the L1 and L2 penalties, the elastic net could minimize some coefficients to zero (L1 penalty) and minimize the value of all coefficients (L2 penalty) simultaneously, and thus applicable to datasets with a limited number of samples (Zou and Hastie 2005).

**Figure 1:**
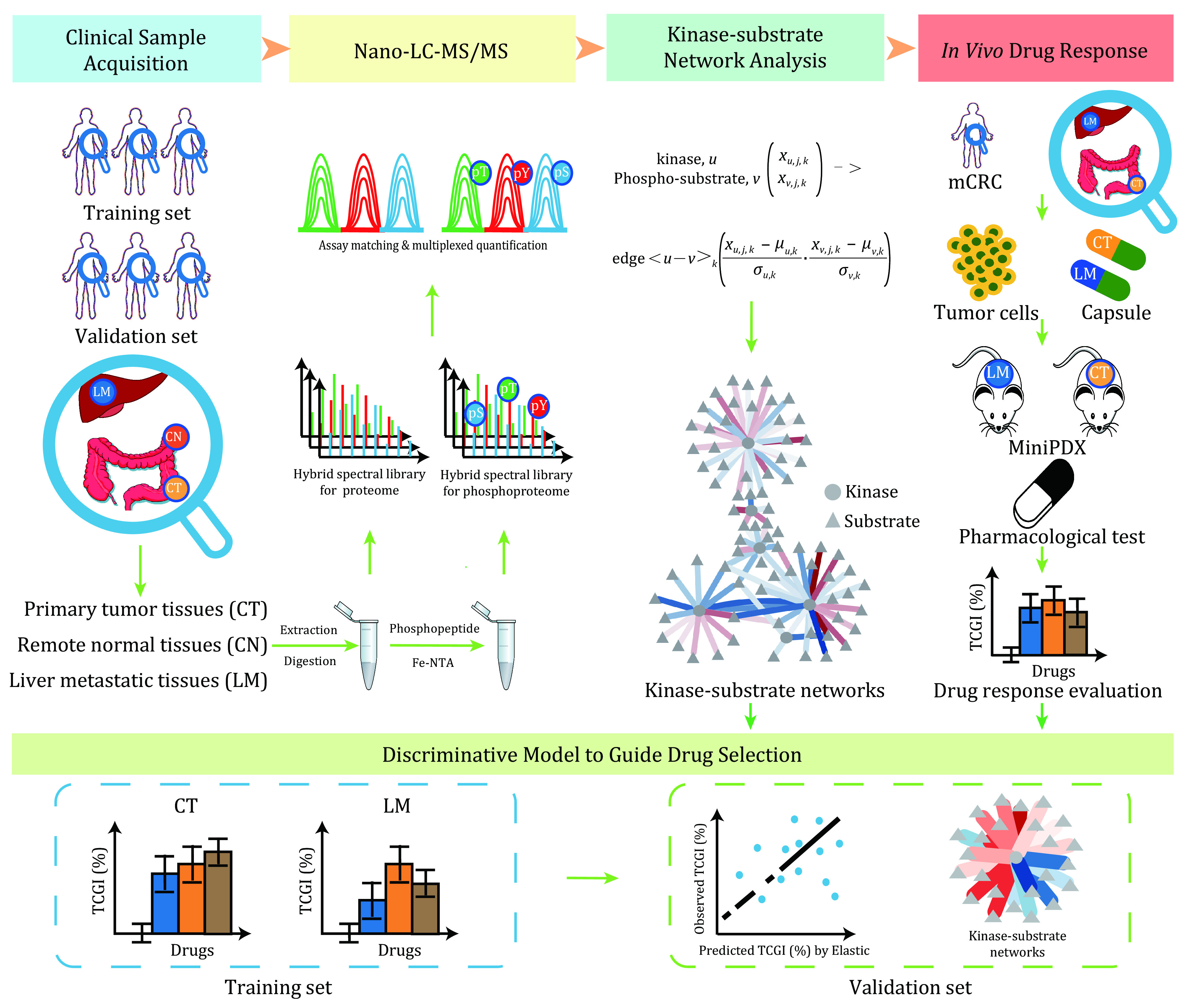
The workflow of DIA mode proteomics and phosphoproteomics to evaluate the personalized drug responses of primary and metastatic tumors in CRC. There were 22 patients in the study, including metastatic CRC and non-metastatic CRC. We collected the patients’ primary tumor tissues, remote normal tissues, and liver metastasis for DIA proteomics and phosphoproteomics analysis. Evaluate personalized drug responses by constructing the kinase-substrate network and using MiniPDX models

Here, we evaluate personalized drug responses in primary and metastatic CRC tumors using DIA proteomic and phosphoproteomic data as well as MiniPDX models. And we provide a workflow from the generation of DIA proteomic and phosphoproteomic data to *in vivo* drug testing models, mainly including clinical sample collection, protein extraction and digestion, phosphopeptide enrichment, high-pH reversed-phase liquid chromatography fractionation, the establishment of hybrid spectral libraries, MS data acquisition, kinase-substrate network construction,* in vivo* drug tests, and elastic regression modeling (Fig. 1).

## MATERIALS AND EQUIPMENT

### Reagents and supplies

• SDT lysis buffer (4% *w*/*v* sodium dodecyl sulfate, SDS, Invitrogen, cat. no. 15525017; 100 mmol/L tris-hydrochloric acid, tris-HCl, Invitrogen, cat. no. 15504020; 0.1 mol/L dithiothreitol, DTT, Sigma, cat. no. D9163-25G; pH 7.6) (Wisniewski* et al.* 2009)

• 50 mmol/L NH_4_HCO_3_ (Sigma, cat. no. A6141)

• Trypsin (Promega, cat. no. V5113)

• High-Select Fe-NTA kit (Thermo Scientific, cat. no. A32992)

• BCA Protein Assay kit (Thermo Scientific, cat. no. 23225)

• Acetonitrile, ACN (Fisher Scientific, cat. no. A955-4)

• Binding/Wash buffer (80% ACN; 0.1% trifluoroacetic acid, TFA, Sigma, cat. no. 302031)

• Methanol (Fisher Scientific, cat. no. A452-4)

• 0.1% Formic acid (Fisher Scientific, cat. no. A117-50)

• Elution buffer (50% ACN; 5% ammonium hydroxide, Sigma, cat. no. 17093)

• Mobile phases A (2% ACN; 20 mmol/L ammonium formate buffer, pH 10)

• Mobile phases B (88% ACN; 20 mmol/L ammonium formate buffer, pH 10)

• Indexed Retention Time (iRT) kit (Biognosys, cat. no. Ki-3002)

• HEK-293T peptide mixtures (Homemade)

• Hank’s balanced salt solution, HBSS (Invitrogen, cat. no. 14170)

• Magnetic beads (Miltenyi Biote, Inc., human anti-CD45 microbeads, cat. no. 130-045-80; human anti-fibroblast microbeads, cat. no. 130-050-601)

• 0.125 mg/mL Collagenase IV (Invitrogen, cat. no. 17104019)

• 0.5% hydroxypropyl methylcellulose, HPMC (Sigma, cat. no. SLBC6147)

• 0.2% Tween 80 (Sigma, cat. no. BCBV0835)

• The CellTiter Glo Luminescent Cell Viability Assay kit (Promega, cat. no. G7571)

• Afatinib (Tsbiochem, cat. no. T1773)

• Gefitinib (Bidepharm, cat. no. BD131918)

• Regorafenib (Bidepharm, cat. no. BD559192)

• Home-packed one-layer Empore-C8 StageTip (Rappsilber* et al.* 2007)

• Home-packed two-layer Empore-C18 StageTip (Rappsilber* et al.* 2007)

• XBridge BEH300 C18 column (250 mm × 4.6 mm, OD 5 mm) (Waters, USA)

• Home-made micro-tip C18 column (75 μm × 200 mm) packed with ReproSil-Pur C18-AQ, 3.0 mm diameter resin (Dr. Maisch GmbH, Germany)

• OncoVee^TM^-MiniPDX capsules (LIDE Biotech, Shanghai, China)

### Equipment

• Shimadzu Prominence HPLC System (Shimadzu, Japan)

• EASY-nLC™ 1000 system (Thermo Fisher Scientific, USA)

### Software

• MaxQuant (Tyanova* et al.* 2016)

• Spectronaut™ (Biognosys)

• R (Version 3.6) (Ihaka and Gentleman 1996)

• PhosphositePlus (Hornbeck* et al.* 2015)

• KSEAapp (Version 0.99.0) (Wiredja* et al.* 2017)

• Cytoscape (Shannon* et al.* 2003)

• Xcalibur™ software (Thermo Fisher Scientific)

## STEP-BY-STEP PROCEDURE

### Step 1: Sign informed consents and collect clinical samples [TIMING 1 d per patient]

We collected written informed consent from all participating CRC patients. Tissue specimens were collected for each patient. In addition to primary tumor tissues (CT), we also collected remote normal tissue (CN, 5-cm-away from the tumor edge), and para-carcinoma tissues (CP, 2-cm-away from the tumor edge, normal adjacent tissues). Metastatic CRC (mCRC) patients also took their hepatectomy of liver metastatic cancer tissues (LM) at the same time. Each tissue specimen was collected within 30 min after resection, immediately transferred into sterile cryovials, immersed in liquid nitrogen, cut into 0.5 cm^3^ pieces under −40 °C, then aliquoted and stored at −80 °C. We summarized the baseline and clinicopathologic features of 22 CRC cases, for building all 31 MiniPDX models, in the supplementary Table S1.

### Step 2: Protein extraction and digestion [TIMING 3 d]

Step 2.1: Tissue specimens were minced, then lysed in SDT lysis buffer followed by 5 min of heating at 95 °C and 3 min of sonication (5 s on and 10 s off, power 50 Watts).

**[TIP]** The ratio of tissue wet-weight to lysate volume is recommended 20 mg tissue to 200 µL SDT lysis buffer.

Step 2.2: The lysate was centrifuged at 14,000 *g* for 10 min to clarify and the supernatant was collected in a new tube for later use.

Step 2.3: Protein concentration was detected using the tryptophan-based fluorescence quantification method (Thakur* et al.*^ 2011^).

Step 2.4: The protein sample was digested by filter-aided sample preparation protocol (FASP) (Wisniewski* et al.* 2009) using 10 kDa centrifugal filter units (Millipore). The initial protein amount of FASP was 200 μg per centrifugal filter. Trypsin (Promega) was added in two rounds in 50 mmol/L NH_4_HCO_3_ solution at 37 °C. The first round lasted 12 h with 1∶50 of total protein amount, and the second round lasted another 4 h with equal amounts of trypsin.

Step 2.5: Each peptide mixture was eluted by centrifugation and dried by speed-vac.

Step 2.6: One-sixth of the collected non-phosphorylated peptides (Step 3.3.5) were desalted on a two-layer C18 homemade StageTip (Rappsilber* et al.* 2007). The clean peptides were lyophilized and resuspended in 0.1% formic acid, and one-tenth was taken for mass spectrometry analysis.

**[TIP]** For each clinical tissue sample, we recommend enzymatic hydrolysis of five centrifugal filters simultaneously. And approximately 500 µg of the peptide will be obtained for the following phosphorylation enrichment step (Step 3).

### Step 3: Phosphopeptide enrichment [TIMING 1 d]

The phosphopeptide enrichment was performed using a High-Select Fe-NTA kit (Thermo Scientific) according to the kit manual and previous report (Gao *et al*. 2019) with some following modifications.

**[TIP]** Equilibrate all solutions to room temperature prior to performing experiments.

#### Step 3.1: Suspend peptide sample

The peptide sample ( ~250 μg) was completely suspended in 200 μL Binding/Wash buffer (80% ACN; 0.1% TFA). Use a vortex mixer with a tube stand if necessary.

**[TIP]** In this step, we recommend pH test trips for trace samples. And the pH of sampling detection should be less than 3.

#### Step 3.2: Equilibrate column

Step 3.2.1: Remove the bottom closure of the spin column and loosen the screw cap.

Step 3.2.2: Place the column in a 2 mL microcentrifuge collection tube. And then centrifuge at 1,000 *g* for 30 s to remove the storage buffer.

Step 3.2.3: Add 200 μL Binding/Wash buffer to the column, centrifuge at 1,000 *g* for 30 s and discard the flowthrough. Repeat this step once.

Step 3.2.4: Cap the bottom of the column with a white Luer plug. And place the column into a new microcentrifuge tube.

#### Step 3.3: Sample loading

Step 3.3.1: Take the resins from the spin column into a microcentrifuge tube. Then add 200 μL Binding/Wash buffer to resins, thoroughly mix and divided into five equal parts, and transfer the resins into a new microcentrifuge tube for later use.

**[TIP]** Thorough mixing is required during dispensing into new microcentrifuge tubes.

Step 3.3.2: Add 40 μL resins into 200 μL suspended peptide sample and mix the sample with resin well.

**[TIP]** We optimized the ratio of peptide and resin, and approximately 40 μL of resins in Binding/Wash buffer was used per 250 μg peptide (Digested from 500 μg initiative protein).

Step 3.3.3: The peptide-resin mixture was incubated for 30 min and interspersed with thrice gentle blowing at room temperature.

Step 3.3.4: Prepare a home-packed one-layer Empore-C8 StageTip (Rappsilber* et al.* 2007). Add 200 μL methanol through the StageTip, and centrifuge at 3,500 *g* for 60 s. And then add 200 μL Binding buffer/Washing buffer into StageTip, centrifuge at 3,500 *g* for 60 s, and repeat once.

Step 3.3.5: Then the peptide-resin mixture was transferred into a home-packed one-layer Empore-C8 StageTip. Centrifuge at 1,000 *g* for 3 min and collect the flowthrough, which is the non-phosphorylated peptides. And then non-phosphorylated peptides were desalted as described above (Step 2.6).

**[TIP]** The speed of centrifugation is required to ensure that the contact between the sample and resin lasts for 3 min. The flowthrough component can be reloaded once.

#### Step 3.4: Wash column

Step 3.4.1: Add 200 μL Binding/Washing buffer to a column, centrifuge at 1,000 *g* for 3 min and discard the flowthrough. Repeat this step two additional times for a total of three washes.

Step 3.4.2: Add 200 μL 50% ACN to a column, centrifuge at 1,000 *g* for 3 min, and discard the flowthrough. Repeat this step once.

#### Step 3.5: Elute column

Step 3.5.1: Place the column in a new microcentrifuge tube. Add 100 μL Elution buffer (50% ACN; 5% ammonium hydroxide) to the column. Centrifuge at 1,000 *g* for 3 min. Repeat this step two additional times for a total of three times.

Step 3.5.2 The eluate was immediately dried by speed-Vac at 45 °C for mass spectrometry analysis.

**[TIP]** During this step, the resin color may change from milky white to brown.

### Step 4: High-pH reversed-phase liquid chromatography (RPLC) fractionation [TIMING 1 d]

Step 4.1: Mobile phases A (2% ACN; 20 mmol/L Ammonium formate buffer, pH 10) and B (88% ACN; 20 mmol/L Ammonium formate buffer, pH 10) were prepared based on the previous paper (Gilar* et al.* 2005).

Step 4.2: Peptide mixtures were fractionated by a Waters XBridge BEH300 C18 column (250 mm × 4.6 mm, OD 5 mm) on Shimadzu Prominence HPLC System following the manufacturer’s instructions.

Step 4.3: As for the proteome spectral library, a 97-min gradient was set as follows, 5%–7.5% B in 2 min; 7.5%–12% B in 5 min; 12%–25% B in 40 min; 25%–32% B in 25 min; 32%–95% B in 7 min; 95% B for 4 min; 95%–5% B in 4 min; 5% B for 10 min. The eluate was auto-collected every 1 min (Except for the last min) into 96 fractions. According to the HPLC chromatogram, 30 fractions for proteome were combined by a concatenation scheme (Song* et al.* 2010). Merging details of 96 offline fractions are given in the supplementary Table S2.

Step 4.4: As for the phosphoproteome spectral library, peptide mixtures digested from 30 mg proteins were used to fractionate ( ~3 mg peptide per time, five times in total). An 85-min gradient was set as follows, 5%–7.5% B in 2 min; 7.5%–12% B in 5 min; 12%–25% B in 35 min; 25%–32% B in 22 min; 32%–95% B in 2 min; 95% B for 4 min; 95%–5% B in 4 min; 5% B for 11 min. The eluate was auto-collected every 2 min into 42 fractions. Next, according to the HPLC chromatogram, 20 fractions were combined by a concatenation scheme (Song* et al.* 2010). Merging details of 42 offline fractions are given in the supplementary Table S2.

Step 4.5: Each fractionation was dried in a speed-Vac and reconstituted to enrich phosphopeptide as described above (Step 3).

**[TIP]** We have tested sequential order for phosphopeptide enrichment and peptide fractionation. The order listed here is the recommended order.

### Step 5: Benchmark for nano-LC-MS/MS [TIMING 0.5 d]

#### Step 5.1: Standard sample preparation

##### Step 5.1.1: iRT-peptides

The iRT-peptide is a mixture of 11 synthetic peptides that are not naturally present. The peptide retention time in the reverse phase chromatography could be converted into iRT space (Escher* et al.* 2012).

##### Step 5.1.2: 1 μg 293T peptide mixture

As for each homemade column for running DDA or DIA raw files, we use accurately quantified 1 μg 293T peptide mixture (BCA Protein Assay Kit, Thermo Scientific) to check the column pressure and column efficiency. HEK-293T cells were grown in DMEM (Gibco) supplemented with 10% fetal bovine serum (FBS) and 1% penicillin-streptomycin. All cells were cultured in a 37 °C incubator with 5% CO_2_. HEK-293T peptide mixtures were obtained by the filter-aided sample preparation protocol (FASP) (Wisniewski* et al.*^ 2009^).

#### Step 5.2: Chromatographic stability checking

We ran a shot of iRT alone to check chromatographic stability.

#### Step 5.3: Sample resolving and separating

The sample was resolved using 0.1% formic acid and was separated using a home-made micro-tip C18 column (75 μm × 200 mm) packed with ReproSil-Pur C18-AQ, 3.0 mm resin (Dr. Maisch GmbH, Germany). Each standard sample was analyzed on a Thermo Scientific™ EASYnLC™ 1000 nanoflow LC.

#### Step 5.4: Parameter setting of LC-MS/MS

Step 5.4.1: Flow rate

300 nL/min with the following gradients:

For iRT peptides, 0–1 min, 10% buffer B (0.1% formic acid in acetonitrile); 1–13 min, 10%–30% B; 13–15 min, 30%–45% B; 15–16 min, 45%–90% B; 16–22 min, 90% B.

For 293T peptide mixture, 0–2 min, 5%–8% buffer B; 2–42 min, 8%–23% B; 42–50 min, 23%–40% B; 50–52 min, 40%–100% B; 52–60 min, 100% B.

Step 5.4.2: Resolution

DDA performed 120K resolution MS scan and then triggered top 20 precursors (QE HF, Thermo Scientific Q Exactive HF hybrid quadrupole-Orbitrap mass spectrometer) for 15K resolution MS/MS scans.

Step 5.4.3: AGC target value and max injection time

The MS or MS/MS AGC target value was set at 3 × 10^6^ with 50 ms or 1 × 10^5^ with 35 ms of max injection time, respectively.

#### Step 5.5: Database searching

For each 293T raw, database searching against the human UniProt database (Download on July 2017) was performed using MaxQuant 1.5.2.8 with default settings (Tyanova* et al.* 2016). The average numbers of identified proteins, identified peptides, and average minute of retention length were around 3200, 17800, and 0.4, respectively.

**[TIP]** It is recommended that, in each day for running DDA or DIA raw files, a shot of iRT peptides alone is the benefit to check chromatographic stability conveniently. The retention time for the first *m*/*z* in iRT peptides was ensured at about 4 ± 0.5 min, and all peak widths of iRT peptides were checked at about 30 s by Xcalibur™ software.

### Step 6: DDA and DIA mode to generate proteomic or phosphoproteomic spectral library [TIMING 75 min MS detection time per DDA or DIA file]

#### Step 6.1: Equal amounts of iRT peptide

Equal amounts of iRT peptide were mixed directly into the MS insert tube of each proteomic or phosphoproteomic peptide mixture.

**[TIP]** The iRT peptide added at this time has minimal loss and will assist in RT time correction during DIA data analysis. From a cost perspective, iRT peptides may not be included.

#### Step 6.2: Equipment

Each DDA or DIA sample was analyzed on a Thermo Scientific™ EASYnLC™ 1000 nanoflow LC. The RP chromatographic column was the same as above (Step 5.3).

#### Step 6.3: Flow rate

The flow rate is 300 nL/min with the following gradients.

For proteome in DDA and DIA mode, 0–2 min, 2%–4% buffer B; 2–58 min, 4%–30% B; 58–66 min, 30%–45% B; 66–69 min, 45%–90% B; 69–75 min, 90% B.

For phosphoproteome in DDA and DIA mode, 0–2 min, 2%–4% buffer B; 2–58 min, 4%–23% B; 58–66 min, 23%–40% B; 66–69 min, 40%–90% B; 69–75 min, 90% B.

#### Step 6.4: Parameter setting of LC-MS/MS

Step 6.4.1: DDA mode

1 A lock-mass *m*/*z* 445.12002 was used for internal calibration.

2 The spray voltage was set at 2,300 V in positive ion mode and the ion transfer tube temperature was set at 270 °C.

3 DDA performed a 120K resolution MS scan @ *m*/*z* 200 and then triggered the top 20 precursors (QE HF). The MS AGC target value was set at 3 × 10^6^ with 50 ms of max injection time by orbitrap mass analyzer (300–1,500 *m*/*z*). The MS/MS AGC target value was set at 1 × 10^5^ with 35 ms of max injection time generated by HCD fragmentation (200–2,000 *m*/*z*) at a resolution of 15,000 @ *m*/*z* 200.

4 The normalized collision energy (NCE) was set at NCE 28%, and the dynamic exclusion time was 30 s.

5 Precursors with charges 1, 7, 8 and > 8 were excluded for MS/MS analysis.

Step 6.4.2: DIA mode

1 Basic parameters were equal to the DDA parameters described above (Step 6.4.1).

2 DIA isolation windows with variable widths were decided by DDA searching results from MaxQuant. First, five proteome and 20 phosphoproteome DDA runs were analyzed by MaxQuant. The distributions of peptide precursors and or phosphopeptide precursors were counted in sections from 300 to 1500. Then, according to the peptide precursor density and or phosphopeptide precursor percentage in each segment, the segments and the isolation window within each segment were divided. Next, about 20 to 30 test DIA raw files were running based on the settings of variable DIA windows, and were rapidly evaluated DIA data quality. In brief, there are three principles for variable DIA method editing: more than six data points need to be used for chromatography peak quantification; if enough data points, more DIA windows, and more narrow isolation widths were recommended; it is necessary to modify the width of each window to make sure each window contains the similar number of precursor ions (Zhang* et al.* 2015).

**[TIP]** This process might take several adjustments.

3 For proteome DIA MS runs, fragment analysis was subdivided into 27 DIA isolation windows of four different widths: 10 loop counts of 29 *m*/*z* with central *m*/*z* at 314.5, 343.5, 372.5, 401.5, 430.5, 459.5, 488.5, 517.5, 546.5, and 575.5; 11 loop counts of 28 *m*/*z* with central *m*/*z* at 604.0, 632.0, 660.0, 688.0, 716.0, 744.0, 772.0, 800.0, 828.0, 856.0, and 884.0; 5 loop counts of 55 *m*/*z* with central *m*/*z* at 925.5, 980.5, 1035.5, 1090.5, and 1145.5; 1 loop count of 300 *m*/*z* with central *m*/*z* at 1323.0. The distribution of peptide precursors in test DDA MS runs and the 27 variable DIA windows for proteome DIA MS runs can be found in more detail in the supplementary Table S3.

4 For phosphoproteome DIA MS runs, fragment analysis was subdivided into 34 DIA isolation windows of five different widths: 2 loop counts of 46 *m*/*z* with central *m*/*z* at 423.0 and 469.0; 4 loop counts of 24 *m*/*z* with central *m*/*z* at 504.0, 528.0, 552.0 and 576.0; 16 loop counts of 19 *m*/*z* with central *m*/*z* at 597.5, 616.5, 635.5, 654.5, 673.5, 692.5, 711.5, 730.5, 749.5, 768.5, 787.5, 806.5, 825.5, 844.5, 863.5 and 882.5; 10 loop counts of 21 *m*/*z* with central *m*/*z* at 902.5, 923.5, 944.5, 965.5, 986.5, 1007.5, 1028.5, 1049.5, 1070.5, and 1091.5; 2 loop counts of 99 *m*/*z* with central *m*/*z* at 1151.5 and 1250.5. The distribution of phosphopeptide precursors in test DDA MS runs and the 34 variable DIA windows for phosphoproteome DIA MS runs can be found in more detail in the supplementary Table S4.

#### Step 6.5: Database searching

Step 6.5.1: DDA files

1 DDA files were processed using MaxQuant (1.6.2.10) with default settings.

2 Carbamidomethyl (C) was set as fixed modifications. Oxidation (M), Acetyl (Protein N-term) were set as variable modifications. In phosphorylation data analysis, phospho (STY) was also set as a variable modification.

3 Reference FASTA files for human was downloaded from UniProt on July 2017, and combined with the fusion sequence of iRT (Biognosys Inc.).

4 A maximum number of five modifications per peptide were allowed for each peptide.

5 Enzyme specificity was set as trypsin/P.

6 The maximum missing cleavage site was set as 2.

7 The tolerances of the first search and main search for peptides were set at 20 ppm and 4.5 ppm, respectively.

8 The minimal peptide length was set at 7.

9 The false discovery rates (FDR) of peptide, protein and site were all < 0.01.

Step 6.5.2: DIA files

1 We used Pulsar to generate spectral libraries from both DDA (MaxQuant results) and DIA files with default settings.

2 Human reference FASTA files were the same as DDA described above (Step 6.5.1).

3 For phosphoproteomic DIA runs, phospho (STY) was also set as a variable modification.

4 False identifications were controlled by an FDR estimation (Cutoff 0.01) at three levels: peptide-spectrum match (PSM), peptide, and protein group level.

### Step 7: Generate hybrid spectral library [TIMING 75 min MS detection time per DDA or DIA file]

We generated a hybrid spectral library using DDA data and DIA data. In order to make the established hybrid spectral library high quality and comprehensive, and can be used multiple times in related scientific research, we used data from four types of CRC tissues (CT, CN, LM and CP) and multiple colorectal cell lines to generate the hybrid spectral library. Project-specific library in DDA mode is created from different cell line samples, tissue samples, tissue sample mixture, and offline pre-fractionated peptide mixtures from pooled individual tissue samples. Meanwhile, high-quality and high-precision spectral libraries were also directly generated from multiple DIA data. The spectral library generated from the DDA files was searched with MaxQuant and built by Spectronaut™, and the directDIA library was generated by Spectronaut™ as described above (Step 6.5). More structural and scale details about the hybrid spectral libraries for proteome and phosphoproteome are given in the supplementary Table S5.

**[TIP]** In this year, Spectronaut™ has been updated to version 16, which can significantly improve both the directDIA analysis and library-based DIA analysis. Therefore, we designed two small experiments to objectively evaluate the capabilities of updated library-free, and library-based DIA analyses. Randomly selected 32 proteomic DIA MS runs and 32 phosphoproteomic DIA MS runs were analyzed by Spectronaut™ 16. The related clinical samples for both experiments were not used to build the project-specific library, but were used to build the directDIA library. The results of proteomic analyses were summarized in the supplementary Fig. S1 and Table S6, while phosphoproteomic analyses were gathered in the supplementary Fig. S2 and Table S7. We can take the following information from the above results. Firstly, although Pan Human Library (Rosenberger* et al.* 2014) has the maximum storage capacity, the database search results are worse than the others (supplementary Table S5 and Fig. S1) because of the less precise. Thus, a resource library should be carefully used, especially for sample sets with large individual variability. Secondly, Spectronaut™ 16 has really improved its library-free ability. Both proteomic and phosphoproteomic directDIA workflows can get much better results at the peptide precursor level and protein group level than the workflows using the directDIA libraries acquired from a large number DIA runs by Spectronaut™ 13. Thirdly, the recovery rates deconvoluted from the directDIA libraries are higher than the other libraries, which indicates that the directDIA libraries present a more prominent precision ability. Fourthly, results showed that the number of identified peptide precursors and protein groups using the hybrid library outperformed other approaches in the two experiments, especially at the phosphopeptide precursor level. Therefore, we suggest that directDIA workflow provides good results without extra MS time for library building using Spectronaut™ 16, DIA-NN 1.8 (Demichev* et al.* 2020), or any other search engines with comparable capabilities. However, as for large sample sets with high biological variance, the hybrid library workflow listed here is still the first choice. The extra time spent deepening the project-specific library in DDA mode and building the precise library for as many individual samples as possible in DIA mode will be worth it.

### Step 8: DIA mode to get proteomic or phosphoproteomic data [TIMING 2 d]

Step 8.1: The nano-LC MS/MS was run as described above in DIA mode (Step 6).

Step 8.2: Set up a DIA analysis in Spectronaut™ 13 and import DIA files to Spectronaut™ 13 software (Step 8.1).

Step 8.3: Assign hybrid libraries, including project-specific library and directDIA library (Step 7, supplementary Table S5), and the protein database is consistent with the spectral library.

Step 8.4: Parameter setting for proteomic data.

Step 8.4.1: Calibration was set to non-linear iRT calibration with precision iRT enabled.

Step 8.4.2: Identification was performed using the 5% *q*-value cutoff on the precursor and the protein level.

Step 8.4.3: The maximum number of decoys was set to a fraction of 0.1 of the library size.

Step 8.4.4: Quantity was determined on MS/MS level using the area of XIC peaks with enabled cross run normalization.

Step 8.5: Parameter setting for phosphoproteomic data.

Step 8.5.1 Minor quantified (Peptide) grouping was set by modified sequence and PTM localization was activated and probability cutoff was set to 0, in order to summarize phosphopeptide or phosphosite later.

Step 8.5.2 Phosphosite quantification was counted from the quantity of phosphorylation sequences by a Perl script according to MaxQuant strategy.

### Step 9: Pre-processing and analysis of proteomic and phosphoproteomic data [TIMING 20 min]

**Figure 2:**
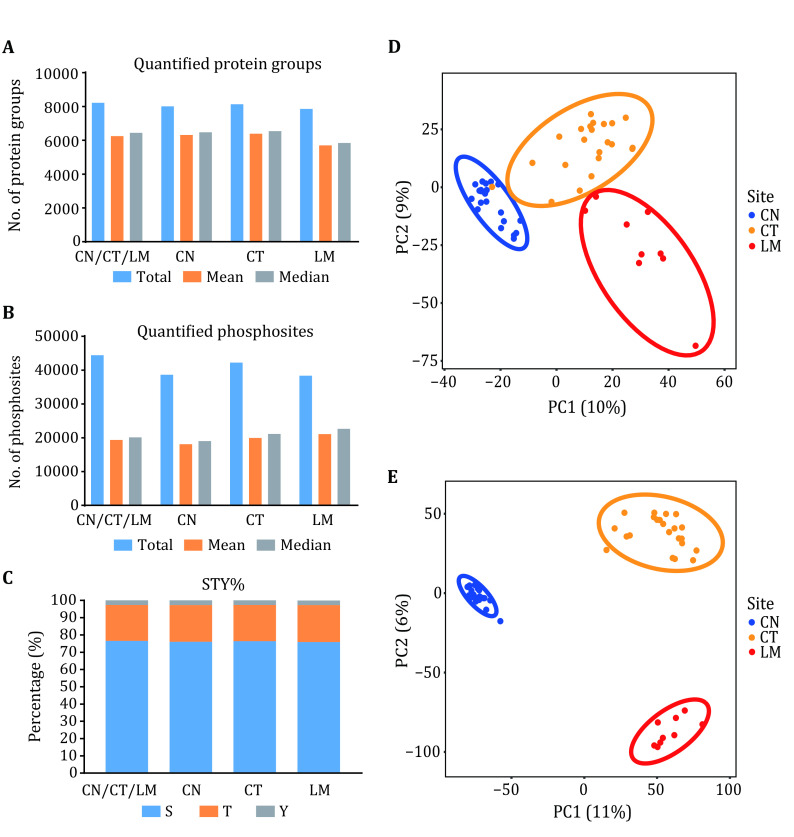
Quality Assessments for MS Data. Details of the proteomic and phosphoproteomic data across all samples or different groups (CN, CT, and LM) are summarized in Proteome (**A**), Phosphoprotein (**B**) and phospho-S/T/Y percentages (**C**). Partial least squares discrimination analysis distinguished CT samples from CN samples and LM samples based on global proteomic data (**D**) and phosphoproteomic data (**E**). CN was also distinguishable from LM in proteomic data (**D**) and phosphoproteomic data (**E**)

Step 9.1: We summarized the proteomic data, phosphoproteomic data, and phospho-S/T/Y percentages for all samples or different groups (CT, CN, LM) (Fig. 2).

Step 9.2: The proteomic and phosphoproteomic data were normalized using the median centering methods and log2-transformed, and then performed partial least squares discrimination analysis (Fig. 2).

### Step 10: Collection of tumor specimens and pretreatment for MiniPDX [TIMING 3 h per clinical tissue sample]

Step 10.1: Fresh surgical tumor specimens were acquired from CRC patients and were washed with Hank’s Balanced Salt Solution (HBSS) to remove mucus and necrotic tumor tissue.

Step 10.2: Tumor tissues were pulverized and digested with collagenase IV for 1–2 h at 37 °C.

Step 10.3: Tumor cells were pelleted by centrifugation at 600 *g* for 5 min.

Step 10.4: The tumor cells were incubated with human anti-CD45 microbeads (cat. No. 130-045-80, Miltenyi Biote, Inc.) and human anti-fibroblast microbeads (cat. No. 130-050-601, Miltenyi Biote, Inc.) at 4 °C for 30 min in the dark, at a final fixed volume of 220 μL, including 20 μL beads, 10^6^ cells and buffer (PBS and 1% FBS). Subsequently, tumor cell suspensions without blood cells or fibroblasts were eluted with 1 mL buffer and collected.

### Step 11: Build MiniPDX model [TIMING 1 h per model]

Step 11.1: The above cell suspension was transferred to HBSS-washed MiniPDX capsules (LIDE Biotech, Shanghai, China).

**[TIP]** Eventually each capsule contained about 2,000 cells.

**Figure 3:**
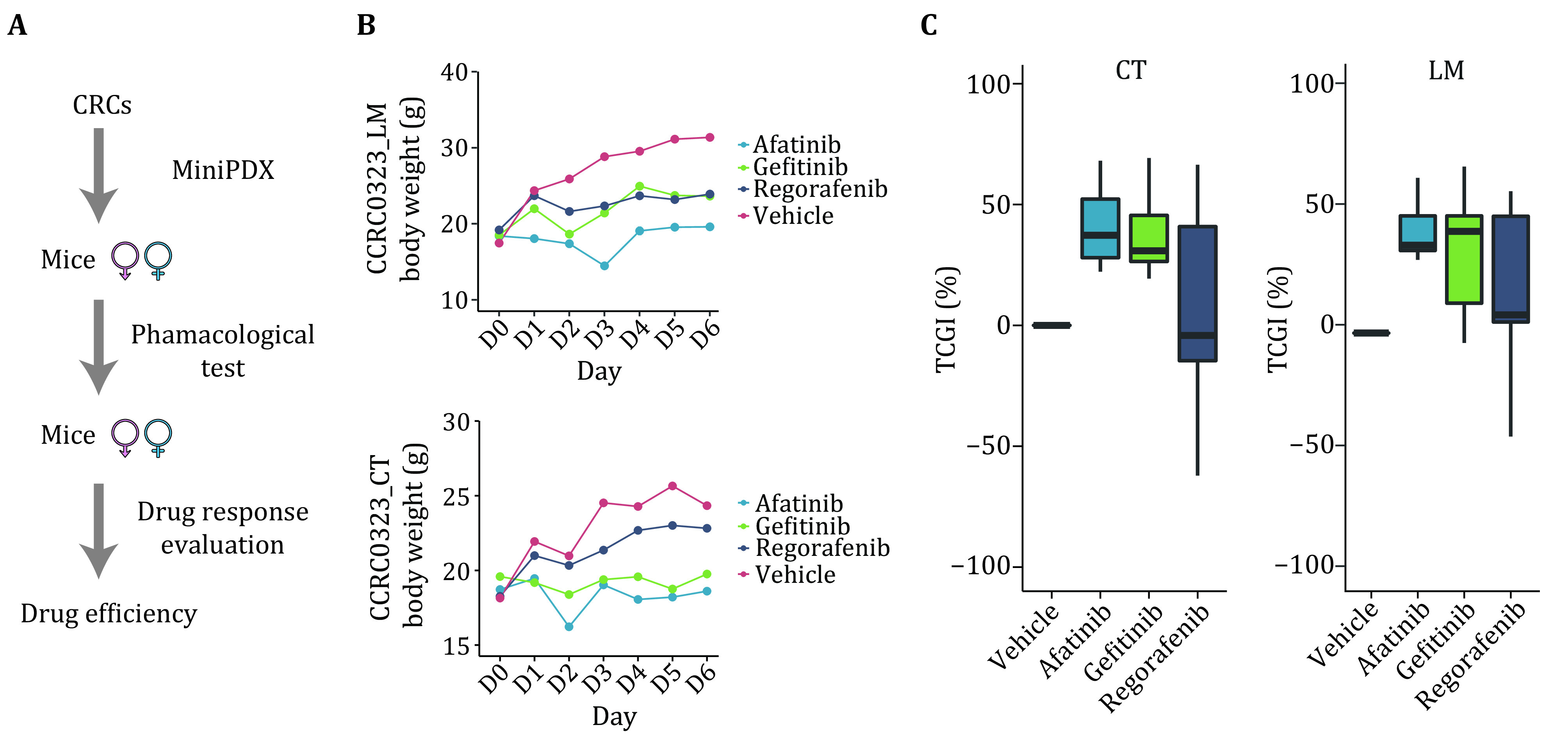
The three drugs’ efficiency was compared in the mice. **A** Schematic diagram of workflow in the mice. **B** The example of mice body weight change in LM and CT mice in three drugs. **C** The efficiency of CT and LM mice in the TCGI (%) was compared

Step 11.2: The capsules were implanted subcutaneously via a small incision in the skin, and each mouse (5-week-old female nu/nu mouse) was implanted with three capsules (Fig. 3A).

### Step 12: Pharmacological test [TIMING 8 d per model]

Step 12.1: Vehicle controls and drugs (Afatinib, gefitinib, or regorafenib) were dissolved by pre-prepared 0.5% hydroxypropyl methylcellulose (HPMC) and 0.2% Tween 80 solutions.

Step 12.2: Following four hours of capsule insertion, mice bearing MiniPDX capsules were treated with appropriate control or drugs (Vehicle controls, afatinib, gefitinib, or regorafenib). Afatinib, gefitinib, and regorafenib were all administered orally, as single administration (Daily [qd] × 1) for continuous seven days with a dose of 20 mg/kg, 75 mg/kg, or 30 mg/kg body weight, respectively. The control group was treated in the same way. Each treatment (Control or drugs) was done in sextuplicate capsules (Fig. 3A).

Step 12.3: Remove all capsules from the mice.

Step 12.4: The proliferation of tumor cells within each capsule was measured using the CellTiter Glo Luminescent Cell Viability Assay kit (G7571, Promega, Madison, WI, US). We also measured weight change in mice over seven days (Fig. 3B, more details are in Fig. S3 in the supplementary information).

Step 12.5: Tumor cell growth inhibition (TCGI) (%) was calculated using the published formula (Zhang* et al.* 2018) (Fig. 3C).

### Step 13: Kinase-substrate network construction [TIMING 15 min]

Step 13.1: We retrieved the kinase and phosphorylation site interaction network from the PhosphositePlus database (Hornbeck* et al.* 2015), where each pair of the experimentally validated relationship between kinase and substrate were reserved.

Step 13.2: To eliminate the influence of tumor heterogeneity in the following analysis, we first normalized the expression levels of both primary tumors and liver metastatic tumors by the protein and phosphorylation site expression levels of remote normal tissues. We divided the expression level of protein or phosphorylation sites of primary tumors and liver metastatic tumors by the value of remote normal tissues, and then made log transformation and gene-level *z*-score transformation for further analysis.

Step 13.3: Calculate the mean and standard deviations for each kinase (protein) and substrate (phosphorylation site) feature in the CT or LM cohort.

Step 13.4: Calculate the edge feature based on the correlation between each pair of kinase (protein) and substrate (phosphorylation site) feature for each sample according to the following formula (Sun* et al.* 2020),

\begin{equation*}\begin{split} &
\begin{array}{c}   \mathrm{k}\mathrm{i}\mathrm{n}\mathrm{a}\mathrm{s}\mathrm{e},u\\    \mathrm{p}\mathrm{h}\mathrm{o}\mathrm{s}\mathrm{p}\mathrm{h}\mathrm{o}{\text{-}}\mathrm{s}\mathrm{u}\mathrm{b}\mathrm{s}\mathrm{t}\mathrm{r}\mathrm{a}\mathrm{t}\mathrm{e},v\end{array}\left(\genfrac{}{}{0pt}{}{{x}_{u,j,k}}{{x}_{v,j,k}}\right)- >\\&
\mathrm{e}\mathrm{d}\mathrm{g}\mathrm{e}{ < u-v > }_{k}\left(\frac{{x}_{u,j,k}-{\mu }_{u,k}}{{{\sigma }}_{u,k}}.\frac{{x}_{v,j,k}-{\mu }_{v,k}}{{\sigma }_{v,k}}\right) ,
\end{split}\end{equation*}

where $ {x}_{u,j,k} $ represents the original value of *u*^th^ kinase in *j*^th^ sample from *k*^th^ class, $ {x}_{v,j,k} $ represents the original value of *v*^th^ phospho-substrate in *j*^th^ sample from *k*^th^ class, and *k* was set to 1 or 2, representing the CT or LM tissue, respectively. In addition, $ {\mu }_{u,k}=\dfrac{1}{{n}_{k}}\displaystyle\sum _{j\;=\;1}^{{n}_{k}}({x}_{u,j,k}-{\mu }_{u,k}) $ and $ {\mu }_{v,k}=\dfrac{1}{{n}_{k}}\displaystyle\sum _{j\;=\;1}^{{n}_{k}}({x}_{v,j,k}-{\mu }_{v,k}) $ are sample means of kinase *u* and phosphor-substrate *v*, and $ {\sigma }_{u,k}=  \sqrt{\dfrac{1}{{n}_{k}}}\displaystyle\sum _{j\;=\;1}^{{n}_{k}}{({x}_{u,j,k}-{\mu }_{u,k})}^{2} $ and $ {\sigma }_{v,k}=\sqrt{\dfrac{1}{{n}_{k}}}\displaystyle\sum _{j\;=\;1}^{{n}_{k}}{({x}_{v,j,k}-{\mu }_{v,k})}^{2} $ are the corresponding uncorrected sample standard deviation.

### Step 14: Elastic net model [TIMING 30 min]

Step 14.1: The 31 MiniPDX models were split into two datasets. The paired 18 models were used as the training dataset, and the other 13 independent models were regarded as testing dataset.

Step 14.2: We first performed a pre-selection step for the kinase and phospho-substrate features based on their Pearson’s correlation coefficients with examined drug sensitivity in the training set.

Step 14.3: Elastic net regression models were built for each drug based on the selected kinase and phosphor-substrate node or edge features. These models were used to predict the drug response of the 13 MiniPDX models in the testing cohort.

Step 14.4: Pearson correlation coefficients were calculated between the predicted drug sensitivity and examined ones to assess the prediction performance.

**Figure 4:**
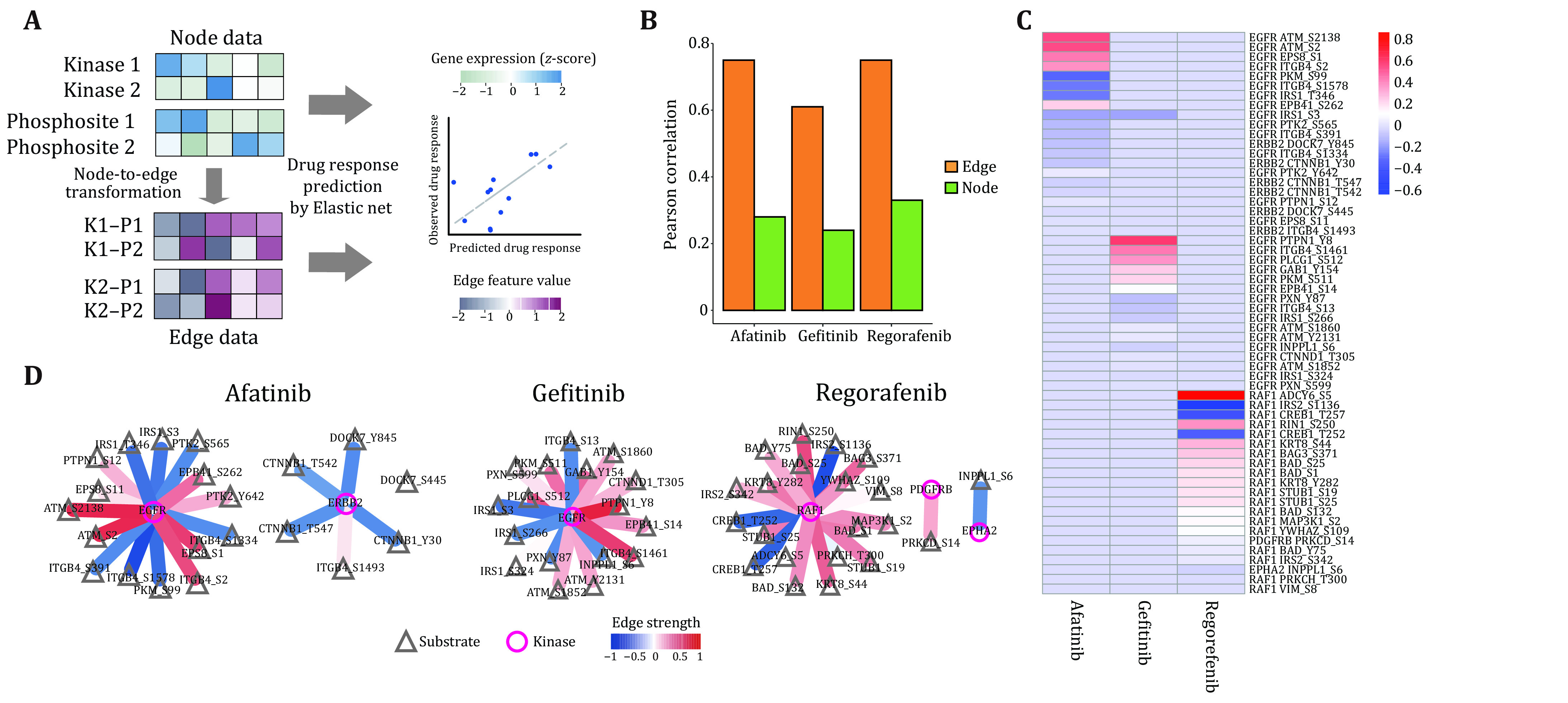
Evaluate the drug responses by constructing the kinase-substrate network and using MiniPDX models. **A** The prediction of Schematic diagram of drug response prediction by proteomics and phosphoproteomics. **B** The Pearson’s correlation between afatinib, gefitinib and regorafenib based on edge and node feature value. The correlation values in the three drugs heatmap (**C**) and connection diagram (**D**)

Step 14.5: Steps 14.2–14.4 were repeated with node features, where the protein expression level of kinases and phosphorylation level of phosphor-substrates were used as input features (Fig. 4).

## ADVANTAGES AND LIMITATIONS OF THE PROTOCOL

This tutorial protocol provides a step-by-step guide for evaluating personalized drug responses in primary and metastatic CRC using DIA proteomic and phosphoproteomic data as well as MiniPDX models. It suggests the potential ability to use proteomic, phosphoproteomic data and kinase-substrate networks to guide drug selection and gives us confidence that we can apply this approach to other disease research areas. In addition, the number of proteins identified by DIA-mode proteomics can be increased by generating a high-quality, comprehensive hybrid spectral library, while it can also be used multiple times in related scientific research. However, generating a high-quality hybrid spectral library is time-consuming, but remains the first choice for DIA proteomics studies in large sample cohorts as well as samples with high biological variability.

## Conflict of interest

Xumiao Li, Yiming Huang, Kuo Zheng, Guanyu Yu, Qinqin Wang, Lei Gu, Jingquan Li, Hui Wang, Wei Zhang, Yidi Sun and Chen Li declare that they have no conflict of interest.

## References

[bAebersold2016] (2016). Mass-spectrometric exploration of proteome structure and function. Nature.

[bBader2020] (2020). Proteome profiling in cerebrospinal fluid reveals novel biomarkers of Alzheimer's disease. Mol Syst Biol.

[bBarkovits2020] (2020). Reproducibility, specificity and accuracy of relative quantification using spectral library-based data-independent acquisition. Mol Cell Proteomics.

[bBertotti2011] (2011). A molecularly annotated platform of patient-derived xenografts ("xenopatients") identifies HER2 as an effective therapeutic target in cetuximab-resistant colorectal cancer. Cancer Discov.

[bBihani2017] (2017). Elacestrant (RAD1901), a selective estrogen receptor degrader (SERD), has antitumor activity in multiple ER(+) breast cancer patient-derived xenograft models. Clin Cancer Res.

[bBilbao2015] (2015). Processing strategies and software solutions for data-independent acquisition in mass spectrometry. Proteomics.

[bBruderer2015] (2015). Extending the limits of quantitative proteome profiling with data-independent acquisition and application to acetaminophen-treated three-dimensional liver microtissues. Mol Cell Proteomics.

[bChen2007] (2007). Differential expression of novel tyrosine kinase substrates during breast cancer development. Mol Cell Proteomics.

[bDemichev2020] (2020). DIA-NN: neural networks and interference correction enable deep proteome coverage in high throughput. Nat Methods.

[bEscher2012] (2012). Using iRT, a normalized retention time for more targeted measurement of peptides. Proteomics.

[bGao2021] (2021). Data-independent acquisition-based proteome and phosphoproteome profiling across six melanoma cell lines reveals determinants of proteotypes. Mol Omics.

[bGao2015] (2015). High-throughput screening using patient-derived tumor xenografts to predict clinical trial drug response. Nat Med.

[bGao2019] (2019). Integrated Proteogenomic Characterization of HBV-Related Hepatocellular Carcinoma. Cell.

[bGilar2005] (2005). Two-dimensional separation of peptides using RP-RP-HPLC system with different pH in first and second separation dimensions. J Sep Sci.

[bGillet2012] Gillet LC, Navarro P, Tate S, Rost H, Selevsek N, Reiter L, Bonner R, Aebersold R (2012) Targeted data extraction of the MS/MS spectra generated by data-independent acquisition: a new concept for consistent and accurate proteome analysis. Mol Cell Proteomics 11(6): O111 016717. https://doi.org/10.1074/mcp.O111.016717

[bGotti2021] (2021). Extensive and accurate benchmarking of DIA acquisition methods and software tools using a complex proteomic standard. J Proteome Res.

[bHochgrfe2010] (2010). Tyrosine phosphorylation profiling reveals the signaling network characteristics of basal breast cancer cells. Cancer Res.

[bHornbeck2015] Hornbeck PV, Zhang B, Murray B, Kornhauser JM, Latham V, Skrzypek E (2015) PhosphoSitePlus, 2014: mutations, PTMs and recalibrations. Nucleic Acids Res 43(Database issue): D512-520

[bIhaka1996] (1996). R: a language for data analysis and graphics. J Comput Graph Stat.

[bKitata2021] (2021). A data-independent acquisition-based global phosphoproteomics system enables deep profiling. Nat Commun.

[bKrasny2021] (2021). Data-independent acquisition mass spectrometry (DIA-MS) for proteomic applications in oncology. Mol Omics.

[bLi2020] (2020). Integrated omics of metastatic colorectal cancer. Cancer Cell.

[bLou2020] (2020). Hybrid spectral library combining DIA-MS data and a targeted virtual library substantially deepens the proteome coverage. iScience.

[bLudwig2018] (2018). Data-independent acquisition-based SWATH-MS for quantitative proteomics: a tutorial. Mol Syst Biol.

[bManley2004] (2004). Advances in the structural biology, design and clinical development of VEGF-R kinase inhibitors for the treatment of angiogenesis. Biochim Biophys Acta.

[bMeierAbt2021] (2021). The protein landscape of chronic lymphocytic leukemia (CLL). Blood.

[bRappsilber2007] (2007). Protocol for micro-purification, enrichment, pre-fractionation and storage of peptides for proteomics using StageTips. Nat Protoc.

[bRicci2014] (2014). Patient-derived ovarian tumor xenografts recapitulate human clinicopathology and genetic alterations. Cancer Res.

[bRosenberger2014] (2014). A repository of assays to quantify 10, 000 human proteins by SWATH-MS. Sci Data.

[bSajic2015] (2015). Using data-independent, high-resolution mass spectrometry in protein biomarker research: perspectives and clinical applications. Proteomics Clin Appl.

[bSchubert2015] (2015). Building high-quality assay libraries for targeted analysis of SWATH MS data. Nat Protoc.

[bShannon2003] (2003). Cytoscape: a software environment for integrated models of biomolecular interaction networks. Genome Res.

[bSong2010] (2010). Reversed-phase-reversed-phase liquid chromatography approach with high orthogonality for multidimensional separation of phosphopeptides. Anal Chem.

[bSpeers2009] (2009). Identification of novel kinase targets for the treatment of estrogen receptor-negative breast cancer. Clin Cancer Res.

[bSun2020] (2020). Kinase-substrate edge biomarkers provide a more accurate prognostic prediction in ER-negative breast cancer. Genomics Proteomics Bioinformatics.

[bSurmen2020] (2020). Phosphoproteomic strategies in cancer research: a minireview. Analyst.

[bThakur2011] Thakur SS, Geiger T, Chatterjee B, Bandilla P, Frohlich F, Cox J, Mann M (2011) Deep and highly sensitive proteome coverage by LC-MS/MS without prefractionation. Mol Cell Proteomics 10(8): M110 003699. https://doi.org/10.1074/mcp.M110.003699

[bTsou2016] (2016). Untargeted, spectral library-free analysis of data-independent acquisition proteomics data generated using Orbitrap mass spectrometers. Proteomics.

[bTyanova2016] (2016). The MaxQuant computational platform for mass spectrometry-based shotgun proteomics. Nat Protoc.

[bWang2021] (2021). Mini-patient-derived xenograft assay based on microfluidic technology promises to be an effective tool for screening individualized chemotherapy regimens for advanced non-small cell lung cancer. Cell Biol Int.

[bWeitsman2014] (2014). Imaging tumour heterogeneity of the consequences of a PKCα–substrate interaction in breast cancer patients. Biochem Soc Trans.

[bWillems2021] (2021). Use of hybrid data-dependent and -independent acquisition spectral libraries empowers dual-proteome profiling. J Proteome Res.

[bWiredja2017] (2017). The KSEA App: a web-based tool for kinase activity inference from quantitative phosphoproteomics. Bioinformatics.

[bWisniewski2009] (2009). Universal sample preparation method for proteome analysis. Nat Methods.

[bZhan2018] (2018). Guided chemotherapy based on patient-derived mini-xenograft models improves survival of gallbladder carcinoma patients. Cancer Commun (Lond).

[bZhang2018] (2018). Characterization of drug responses of mini patient-derived xenografts in mice for predicting cancer patient clinical therapeutic response. Cancer Commun (Lond).

[bZhang2015] (2015). The use of variable Q1 isolation windows improves selectivity in LC-SWATH-MS acquisition. J Proteome Res.

[bZhao2018] (2018). Personalized treatment based on mini patient-derived xenografts and WES/RNA sequencing in a patient with metastatic duodenal adenocarcinoma. Cancer Commun (Lond).

[bZou2005] (2005). Regularization and variable selection via the elastic net. J R Statist Soc B.

